# Influences on and Measures of Unintentional Group Synchrony

**DOI:** 10.3389/fpsyg.2016.01744

**Published:** 2016-11-09

**Authors:** Melissa Ellamil, Josh Berson, Daniel S. Margulies

**Affiliations:** Max Planck Research Group for Neuroanatomy & Connectivity, Max Planck Institute for Human Cognitive and Brain SciencesLeipzig, Germany

**Keywords:** group synchrony, interpersonal coordination, collective experience, group processes, social interaction

## Abstract

Many instances of large-scale coordination occur in real-life social situations without the explicit awareness of the individuals involved. While the majority of research to date has examined dyadic interactions – those between two individuals – during intentional or deliberate coordination, the present review surveys the handful of recent studies investigating behavioral and physiological synchrony across groups of more than two people when coordination was not an explicit goal. Both minimal (e.g., visual information, shared location) and naturalistic (e.g., choir voice section, family relationship) group interactions appear to promote unintentional group synchrony although they have so far only been studied separately. State differences in unintentional group synchrony, or the relative presence of coordination in various conditions, have tended to be assessed differently, such as using correlation-type relationships, compared to its temporal dynamics, or changes over time in the degree of coordination, which appear to be best captured using phase differences. Simultaneously evaluating behavioral, physiological, and social responses as well systematically comparing different synchrony measures could further our understanding of the influences on and measures of group synchrony, allowing us to move away from studying individual persons responding to static laboratory stimuli and toward investigating collective experiences in natural, dynamic social interactions.

## Introduction

Interpersonal synchrony, or the temporal coordination of actions and responses among individuals, is a common and central feature of human interactions and gatherings. Coordinated movement is theorized to have evolved to establish and maintain group cohesion, promoting enhanced coordination during survival-relevant activities such as hunting or warfare (McNeill, 1997; Phillips-Silver et al., 2010; Dunbar, 2012). Psychological experiments have shown that synchrony of movement between different individuals appears to blur the boundary between self and other, fostering social rapport and increasing group cooperation (Hove and Risen, 2009; Wiltermuth and Heath, 2009; Lakens and Stel, 2011). Similarly, anthropological field studies have observed that physical synchrony during large-scale social gatherings, such as Andaman Islanders performing dance rituals (Radcliffe-Brown, 1922), street revelers during Carnival (Ehrenreich, 2007), and ravers dancing to beat-heavy music (Olaveson, 2004), leads to feelings of being one with the community or “collective effervescence” (Durkheim, 1915). However, while interpersonal synchrony appears to both reflect and influence social processes, the factors that give rise to this phenomenon and the means by which it can be quantified have only recently become a topic of increased research interest.

This has been in part due to difficulties in operationalizing the construct of interpersonal synchrony (Bernieri and Rosenthal, 1991). Some researchers have described synchrony as the correspondence between two or more individuals’ internal states and attitudes (Scheflen, 1964). Others have described synchrony instead as the correspondence between two or more individuals’ endogenous behavioral rhythms, such as verbal or bodily activity cycles (Davis, 1982). Correspondence between people’s internal states and endogenous rhythms, however, is often thought to be reflected in the degree of temporal congruence in their physical behaviors (Scheflen, 1964; Davis, 1982; Bernieri and Rosenthal, 1991). Consequently, synchrony has also been defined as simply the co-occurrence in time of two or more individuals’ behaviors, including muscle movements, body postures, and vocalizations (Condon and Ogston, 1966).

Although joint action, communication, and relationship formation rely on the coordination of behaviors between two individuals (Tickle-Degnen and Rosenthal, 1990; Fiske, 1992; Clark, 1996), such coordination also occurs and is frequently required in situations involving three or more people. However, while a large body of research has attempted to examine and quantify unintentional and intentional dyadic synchrony (see Marsh et al., 2009; Oullier and Kelso, 2009 for reviews), it remains unclear if or how such investigations and measures could be generalized to the study of group synchrony (Richardson et al., 2012), which may underlie other important social processes such as group cohesiveness (Campbell, 1958) and group decision making (Janis, 1982). In addition, while joint social activities often require individuals to intentionally or deliberately coordinate their behaviors to achieve a shared goal (see Keller et al., 2014; Elliott et al., 2016 for reviews), many social interactions that do not instruct individuals to align their behaviors also frequently lead to unintentional or spontaneous coordination (Richardson et al., 2007; Lakens, 2010). In contrast to intentional synchrony, which tends to override the intrinsic dynamics or rhythms of individuals’ behaviors, unintentional synchrony enables the assessment of the factors that constrain and maintain coordination among people’s movements or responses during unstructured interactions (Richardson et al., 2007; Lakens, 2010).

Early experimental work, which relied on behavior coding by trained observers at regular intervals, showed unintentional coordination of a group of listeners’ non-verbal behaviors like nodding and posture to a speaker’s speech and movements (e.g., Kendon, 1970; LaFrance and Broadbent, 1976; Hadar, 1989; see Bernieri and Rosenthal, 1991 for review). The present review surveys the handful of more recent studies that have examined synchrony of behavioral and physiological responses across more than two people when coordination was not an explicit goal outside of listener-speaker interactions, providing an overview of the research questions addressed and the methods of quantifying synchrony employed.

## Influences On Synchrony

Previous studies of unintentional group synchrony have investigated how shared experiences, or common contexts and situations, are reflected in behavioral and physiological responses. We divide these studies into two categories based on the level of contextual constraint in the experimental design: minimal group interaction and naturalistic group interaction. Minimal group interaction involves incidental mutual environmental and sensory information (e.g., visual information, shared location) and has been associated with increased movement synchrony. On the other hand, naturalistic group interaction reflects some form of organizational or social structure (e.g., choir voice section, family relationship) and has been found to correspond with enhanced autonomic synchrony.

### Minimal Group Interaction

Some studies of unintentional group synchrony have examined the influence of minimal group interaction. Such experiments are designed to involve the least possible reciprocal action between individuals following from incidental mutual sensory information. These studies investigated whether simply being in a room together with other participants or having visual information on other participants was enough to promote group coordination. These paradigms provide a constrained experimental setting to investigate variables that contribute to unintentional group synchrony, although they may not necessarily be representative of real-life group interactions.

In a laboratory setting with minimal interaction, Richardson et al. (2012) examined if group synchrony could be facilitated by mutual information among individuals. Groups of six participants each rocked chairs while seated in a circle facing the center, either with their eyes closed, which would have captured chance coordination, or with their eyes open, which provided information about others’ actions. Rocking chair movements showed higher synchrony among participants when their eyes were open, and thus had visual information about other group members, compared to when their eyes were closed.

Similarly, Codrons et al. (2014) assessed the influence of other people’s presence on movement and autonomic coordination across individuals in the absence of explicit cues to synchronize. Groups of 10 participants each performed an arm lifting motion without instructions on whether to synchronize to an external auditory rhythm (i.e., metronome beat or music excerpt). The participants performed the experiment either one at a time, which controlled for spurious coordination due to a movement intervention, or together at the same time, which tested for the effect of group settings. Participants assessed collectively in a group, compared to those examined individually, displayed greater synchrony of arm swinging during both the metronome-assisted and uncued conditions. They also displayed greater synchrony of breathing time courses measured by a respiratory belt during the music-associated and rest conditions.

### Naturalistic Group Interaction

Other studies of unintentional group synchrony, in contrast, have investigated the effects of naturalistic group interaction. These experiments evaluate individuals as they perform real-world tasks or participate in real-world events that involve a predetermined social or cultural structure. More ecologically valid contexts such as these, while limited in the types of causal inferences that can be drawn, enable researchers to study the dynamics of unintentional synchrony in real-life group interactions.

In a laboratory study with naturalistic interaction, Müller and Lindenberger (2011) examined whether intentional behavioral coordination among individuals, such as singing in a choir, also promotes unintentional synchrony of autonomic responses. Cardiac and respiratory measures from eleven singers and one conductor were obtained during performance of a music piece either in multiple voice parts or all in unison. Choir members demonstrated higher heart rate and breathing synchrony during singing relative to resting and when singing together compared to singing different voice parts. Breathing synchrony may have arisen as a function of note duration since holding the same pitch for the same period would require similar inhalation and exhalation patterns for individuals singing together. Breathing synchrony, in turn, may have facilitated heart rate synchrony since inhalation has been associated with increased heart rate and exhalation with decreased heart rate (Schäfer et al., 1998; Yasuma and Hayano, 2004).

Outside of a laboratory setting, during a fire walking ceremony, Konvalinka et al. (2011) assessed the effects of ritual participation and observation on autonomic synchrony, in the absence of coordinated movement, in a group with various degrees of relatedness. Arousal, as indexed by heart rate, was measured in 38 individuals belonging to different groups: fire walk participants, local and related spectators, and non-local and non-related spectators. Even with the lack of overt synchronized behavior, active participants and related spectators showed higher synchrony of heart rate compared to unrelated fire walkers and audience members. Heart rate synchrony may have been promoted by similar arousal increases and decreases during the fire walking ritual between performers and related spectators. These common arousal dynamics may have resulted from shared or mirrored emotions (Jackson et al., 2006; Maughan and Gleeson, 2008) enhanced by the presence of a close relationship (Hein and Singer, 2008).

In addition, Néda et al. (2000a) investigated the development and dynamics of coordinated rhythmic applause in concert halls. They recorded audiences at several theater and opera performances using microphones hanging from the ceiling as well as near some randomly selected individuals. Clapping by audience members exhibited greater synchrony during slower clapping rhythms with less variability across individuals relative to faster clapping rhythms with more variability across individuals. There is a lack of synchronization during early applause as audience members clap faster to make noise and show appreciation. Slower, and thus quieter, applause then develops as they slow or double their clapping period in an inadvertent attempt to synchronize with others. Synchronization disappears as the applause continues when they slip back to fast clapping to increase noise again.

The small number of studies conducted so far and the limited types of measurements collected, however, make it difficult to draw conclusions about how shared experiences influence unintentional group synchrony of behavioral and physiological responses. Does minimal group interaction promote physiological synchrony to the same degree as naturalistic group interaction? Does behavioral synchrony arising from shared experience facilitate physiological synchrony or vice versa? Do behavioral and physiological synchrony resulting from shared experience contribute differentially to social bonding? These questions could be more precisely addressed by simultaneously evaluating behavioral, physiological, and social responses.

## Measures Of Synchrony

Prior studies on unintentional group synchrony have developed various ways of quantifying behavioral and physiological responses corresponding with shared experiences. We categorize these methods into two types based on the aspect of interpersonal coordination they evaluate: state differences and temporal dynamics. Our review indicates that state differences in group synchrony, reflecting comparisons between particular conditions, have been mostly described using correlation. In contrast, the temporal dynamics of group synchrony, which capture similarity or dissimilarity over time, seem to be better represented by phase difference.

### State Differences

Several studies of unintentional group synchrony compared movement or autonomic coordination among individuals between different group or time conditions. These state differences in group synchrony, which are reduced to a single index of similarity for an entire time period, have been assessed for the most part using correlation- or coherence-type relationships between each pair of participant time courses. In addition, while some of these methods simply calculate an index of similarity among individuals’ behavioral or physiological responses, other methods extract additional indices that describe other aspects of the relationship among individuals.

With generalized partial directed coherence (Schelter et al., 2009; Codrons et al., 2014), a partial coherence spectrum plot, which represents the degree of similarity between time courses as a function of frequency while controlling for the influence of other participants, is first created for each pair of participants. Coherence values are then averaged for each plot (i.e., pair) and in turn averaged across all pairs for each condition. Similarly, intersubject correlation analysis (Hasson et al., 2004; Kauppi et al., 2014) first computes a correlation coefficient for each pair of participants as a measure of similarity between their behavioral or physiological time courses. These correlation coefficients are then averaged across all pairs for each condition.

Meanwhile, in cross-recurrence quantification analysis (Webber and Zbilut, 1994; Shockley et al., 2002; Konvalinka et al., 2011), a cross-recurrence plot, which represents every instance when one phase space trajectory goes through the same region as another phase space trajectory (i.e., when a point along a time course has the same value as a point along another time course), is constructed for each pair of participants. Different indices for each plot (i.e., pair), such as predictability (i.e., strength of coupling) and stability (i.e., duration of coupling), are then calculated and entered in a comparison of means between conditions. In contrast, phase synchronization indices (Kitzbichler et al., 2009; Müller and Lindenberger, 2011) are derived from a matrix where each row represents the phase difference over time between one pair of participants’ behavioral or physiological time courses. For each row (i.e., pair), indices denoting the coupling duration (i.e., how long phase differences remained constant), overall coupling (i.e., proportion of in-phase synchronization), and positive or negative coupling (i.e., deviations from complete in-phase synchronization) are computed and used in a comparison of means between conditions.

### Temporal Dynamics

In contrast, a few studies of unintentional group synchrony instead examined how movement or autonomic coordination across individuals developed over time. These measures of the temporal dynamics of group synchrony, which are derived from some index of similarity at each time point, have tended to be based on phase differences between participants’ time courses. Some of these methods calculate an index of similarity using pairwise statistics, while other methods derive an index of similarity from group information.

To assess time-varying group synchrony, intersubject phase synchronization and sliding window intersubject correlation average across measures of similarity over time between each pair of participants in a group. With intersubject phase synchronization (Rosenblum et al., 1996; Glerean et al., 2012; Kauppi et al., 2014), the instantaneous phase time series for each participant’s behavioral or physiological response is first extracted. The difference at each time point between the time series of each pair of participants is then calculated as an indicator of their similarity. Finally, the difference time courses from all pairs of participants are averaged and normalized such that higher values represent greater group synchrony. With sliding window intersubject correlation (Glerean et al., 2012; Kauppi et al., 2014), correlations between each pair of participants’ time series are calculated for multiple time points within a sliding window, representing similarity between two individuals’ responses over time. The time courses of correlation values from all pairs of participants are then averaged, with higher values indicating greater group synchrony. Thus, these measures define group similarity as the aggregate of pairwise interactions, which enables the relatively straightforward generalization of established methods for evaluating dyadic coordination to investigations of group synchrony.

In contrast, other ways of assessing time-varying group synchrony, such as the cluster phase method and Kuramoto order parameter, calculate a measure of similarity over time based on information from the group as a whole. With the cluster phase method (Frank and Richardson, 2010; Richardson et al., 2012), the instantaneous phase time series for each participant’s behavioral or physiological response is first extracted and then averaged across all participants to obtain one group time series. The difference at each time point between each individual time series and the group time series is then calculated as an indicator of a participant’s similarity to the group. Finally, the difference time courses from all participants are averaged and normalized such that higher values represent greater group synchrony. With the Kuramoto order parameter (Néda et al., 2000a,b), only an overall group time series (i.e., not from individual data) for a particular response (e.g., the noise intensity of applause after a performance) is used to compute a measure of similarity among individual participants. First, a short-time moving-average time course (e.g., the square of the original clapping noise signal relative to the mean level) is constructed from the group time series. The order parameter, which is the maximum of the normalized correlation between the resulting time course and a harmonic function, is then calculated at each time point, with higher values indicating greater group synchrony. Thus, these measures define group similarity as the combination of individual responses relative to the group, which may allow a more direct and intuitive characterization of group synchrony compared to combined pairwise interactions.

In general, there does not seem to be a standard method for quantifying unintentional group synchrony. Are similarities between individuals’ behavioral or physiological responses better captured by correlation or coherence, which is based on variance information or data spread, or by phase difference, which is based on temporal information or timing details? Are similarities amongst individuals’ behavioral or physiological responses better represented by correspondence between pairs of individuals or by the relationship of one individual to the rest of the group? Future studies would greatly benefit from a systematic comparison of the discriminability and reliability of these different methods of quantifying unintentional group synchrony.

## Conclusion

Unintentional group synchrony of behavioral and physiological responses is an essential feature of numerous social processes. Findings so far suggest that minimal (e.g., visual information, shared location) and naturalistic (e.g., choir voice section, family relationship) group relationships both promote unintentional group synchrony of body movements and autonomic responses. In addition, state differences in unintentional group synchrony have been primarily evaluated using correlation- or coherence-type relationships between participants. On the other hand, the temporal dynamics of unintentional group synchrony have been mostly assessed using phase differences between participants as they capture moment-by-moment changes.

However, the limited number of studies that have examined unintentional group synchrony and the lack of standardized methods for measuring it mean that the factors that influence it and the means by which it can be quantified remain unclear. Do minimal and naturalistic group relationships, as well as behavioral and physiological synchrony, contribute differentially to social processes? Which aspects of similarity between individuals’ responses are accurately and reliably captured by correlations or coherences compared to phase differences and by pairwise statistics compared to group-based calculations?

Understanding how these influences on and measures of unintentional group synchrony reflect social processes is crucial as we move away from studying individual persons responding to static laboratory stimuli and toward investigating collective experiences in natural, dynamic social interactions. Future studies could address these outstanding questions by evaluating behavioral, physiological, and social responses concurrently. Finally, a systematic evaluation of the different measures available would provide valuable insights regarding how unintentional group synchrony could be quantified.

## Author Contributions

ME, JB, and DSM contributed to the conception and writing of the content for the review.

## Conflict of Interest Statement

The authors declare that the research was conducted in the absence of any commercial or financial relationships that could be construed as a potential conflict of interest.

## References

[B1] Bernieri F. J. Rosenthal R. (1991). “Interpersonal coordination: behavior matching and interactional synchrony,” in *Fundamentals of Nonverbal Behavior*, eds Feldman R. S. Rimé B. (New York, NY: Cambridge University Press), 401–432.

[B2] Campbell D. T. (1958). Common fate, similarity, and other indices of the status of aggregates of persons as social entities. *Behav. Sci.* 3 14–25. 10.1002/bs.3830030103

[B3] Clark H. H. (1996). *Using Language.* Cambridge: Cambridge University Press.

[B4] Codrons E. Bernardi N. F. Vandoni M. Bernardi L. (2014). Spontaneous group synchronization of movements and respiratory rhythms. *PLoS ONE* 9:e107538 10.1371/journal.pone.0107538 PMC4162643 25216280

[B5] Condon W. S. Ogston W. D. (1966). Sound film analysis of normal and pathological behavior patterns. *J. Nerv. Ment. Dis.* 143 338–347. 10.1097/00005053-196610000-00005 5958766

[B6] Davis M. E. (1982). *Interaction Rhythms: Periodicity in Communicative Behavior*. New York, NY: Human Sciences Press.

[B7] Dunbar R. (2012). “On the evolutionary function of song and dance,” in *Music, Language, and Human Evolution*, ed. Bannan N. (Oxford: Oxford University Press), 201–214.

[B8] Durkheim E. (1915). *The Elementary Forms of the Religious Life.* London: George Allen and Unwin.

[B9] Ehrenreich B. (2007). *Dancing in the Streets: A History of Collective Joy*. New York, NY: Metropolitan Books.

[B10] Elliott M. T. Chua W. L. Wing A. M. (2016). Modelling single-person and multi-person event-based synchronisation. *Curr. Opin. Behav. Sci.* 8 167–174. 10.1016/j.cobeha.2016.01.015

[B11] Fiske A. P. (1992). The four elementary forms of sociality: framework for a unified theory of social relations. *Psychol. Rev.* 99 689–723. 10.1037/0033-295X.99.4.689 1454904

[B12] Frank T. D. Richardson M. J. (2010). On a test statistic for the Kuramoto order parameter of synchronization: an illustration for group synchronization during rocking chairs. *Physica D* 239 2084–2092. 10.1016/j.physd.2010.07.015

[B13] Glerean E. Salmi J. Lahnakoski J. M. Jääskeläinen I. P. Sams M. (2012). Functional magnetic resonance imaging phase synchronization as a measure of dynamic functional connectivity. *Brain Connect.* 2 91–101. 10.1089/brain.2011.0068 22559794 PMC3624768

[B14] Hadar U. (1989). Two types of gesture and their role in speech production. *J. Lang. Soc. Psychol.* 8 221–228. 10.1177/0261927X8983004

[B15] Hasson U. Nir Y. Levy I. Fuhrmann G. Malach R. (2004). Intersubject synchronization of cortical activity during natural vision. *Science* 303 1634–1640. 10.1126/science.1089506 15016991

[B16] Hein G. Singer T. (2008). I feel how you feel but not always: the empathic brain and its modulation. *Curr. Opin. Neurobiol.* 18 153–158. 10.1016/j.conb.2008.07.012 18692571

[B17] Hove M. J. Risen J. L. (2009). It’s all in the timing: interpersonal synchrony increases affiliation. *Soc. Cogn.* 27:949 10.1521/soco.2009.27.6.949

[B18] Jackson P. L. Rainville P. Decety J. (2006). To what extent do we share the pain of others? Insight from the neural bases of pain empathy. *Pain* 125 5–9. 16997470 10.1016/j.pain.2006.09.013

[B19] Janis I. L. (1982). *Groupthink: Psychological Studies of Policy Decisions and Fiascoes*. Boston, MA: Houghton Miﬄin.

[B20] Kauppi J.-P. Pajula J. Tohka J. (2014). A versatile software package for inter-subject correlation based analyses of fMRI. *Front. Neuroinform.* 8:2 10.3389/fninf.2014.00002 PMC3907702 24550818

[B21] Keller P. E. Novembre G. Hove M. J. (2014). Rhythm in joint action: psychological and neurophysiological mechanisms for real-time interpersonal coordination. *Philos. Trans. R. Soc. Lond. B Biol. Sci.* 369 20130394 10.1098/rstb.2013.0394 PMC4240961 25385772

[B22] Kendon A. (1970). Movement coordination in social interaction: some examples described. *Acta Psychol. (Amst.)* 32 101–125. 10.1016/0001-6918(70)90094-6 5444439

[B23] Kitzbichler M. G. Smith M. L. Christensen S. R. Bullmore E. (2009). Broadband criticality of human brain network synchronization. *PLoS Comput. Biol.* 5:e1000314 10.1371/journal.pcbi.1000314 PMC2647739 19300473

[B24] Konvalinka I. Xygalatas D. Bulbulia J. Schjødt U. Jegindø E.-M. Wallot S. (2011). Synchronized arousal between performers and related spectators in a fire-walking ritual. *Proc. Natl. Acad. Sci. U.S.A.* 108 8514–8519. 10.1073/pnas.1016955108 21536887 PMC3100954

[B25] LaFrance M. Broadbent M. (1976). Group rapport: posture sharing as a nonverbal indicator. *Group Organ. Manag.* 1 328–333. 10.1177/105960117600100307

[B26] Lakens D. (2010). Movement synchrony and perceived entitativity. *J. Exp. Soc. Psychol.* 46 701–708. 10.3389/fphys.2012.00405

[B27] Lakens D. Stel M. (2011). If they move in sync, they must feel in sync: movement synchrony leads to attributions of rapport and entitativity. *Soc. Cogn.* 29 1–14. 10.1521/soco.2011.29.1.1

[B28] Marsh K. L. Richardson M. J. Schmidt R. C. (2009). Social connection through joint action and interpersonal coordination. *Top. Cogn. Sci.* 1 320–339. 10.1111/j.1756-8765.2009.01022.x 25164936

[B29] Maughan R. J. Gleeson M. (2008). Heart rate and salivary cortisol responses in armchair football supporters. *Med. Sport.* 12 20–24. 10.2478/v10036-008-0004-z

[B30] McNeill W. H. (1997). *Keeping Together in Time.* Cambridge: Harvard University Press.

[B31] Müller V. Lindenberger U. (2011). Cardiac and respiratory patterns synchronize between persons during choir singing. *PLoS ONE* 6:e24893 10.1371/journal.pone.0024893 PMC3177845 21957466

[B32] Néda Z. Ravasz E. Brechet Y. Vicsek T. Barabási A.-L. (2000a). Self-organizing processes: the sound of many hands clapping. *Nature* 403 849–850. 10.1038/35002660 10706271

[B33] Néda Z. Ravasz E. Vicsek T. Brechet Y. Barabási A.-L. (2000b). Physics of the rhythmic applause. *Phys. Rev. E* 61:6987 10.1103/PhysRevE.61.6987 11088392

[B34] Olaveson T. (2004). “‘Connectedness’ and the rave experience: rave as new religious movement?,” in *Rave, Culture, and Religion*, ed.John G.St. (New York, NY: Routledge), 85–106.

[B35] Oullier O. Kelso J. A. (2009). “Social coordination from the perspective of coordination dynamics,” in *Encyclopedia of Complexity and Systems Science*, ed.Meyers R. A. (Berlin: Springer-Verlag), 8198–8213.

[B36] Phillips-Silver J. Aktipis C. A. Bryant G. A. (2010). The ecology of entrainment: foundations of coordinated rhythmic movement. *Music Percept.* 28 3–14. 10.1525/mp.2010.28.1.3 21776183 PMC3137907

[B37] Radcliffe-Brown A. R. (1922). *The Andaman Islanders: A Study in Social Anthropology.* London: Cambridge University Press.

[B38] Richardson M. J. Garcia R. L. Frank T. D. Gergor M. Marsh K. L. (2012). Measuring group synchrony: a cluster-phase method for analyzing multivariate movement time-series. *Front. Physiol.* 3:405 10.3389/fphys.2012.00405 PMC3475977 23091463

[B39] Richardson M. J. Marsh K. L. Isenhower R. W. Goodman J. R. Schmidt R. C. (2007). Rocking together: dynamics of intentional and unintentional interpersonal coordination. *Hum. Mov. Sci.* 26 867–891. 10.1016/j.humov.2007.07.002 17765345

[B40] Rosenblum M. G. Pikovsky A. S. Kurths J. (1996). Phase synchronization of chaotic oscillators. *Phys. Rev. Lett.* 76 1804–1807. 10.1103/PhysRevLett.76.1804 10060525

[B41] Schäfer C. Rosenblum M. G. Kurths J. Abel H.-H. (1998). Heartbeat synchronized with ventilation. *Nature* 392 239–240. 10.1038/32567 9521318

[B42] Scheflen A. E. (1964). The significance of posture in communication systems. *Psychiatry* 27 316–331. 14216879 10.1080/00332747.1964.11023403

[B43] Schelter B. Timmer J. Eichler M. (2009). Assessing the strength of directed influences among neural signals using renormalized partial directed coherence. *J. Neurosci. Methods* 179 121–130. 10.1016/j.jneumeth.2009.01.006 19428518

[B44] Shockley K. Butwill M. Zbilut J. P. Webber C. L. (2002). Cross recurrence quantification of coupled oscillators. *Phys. Lett. A* 305 59–69. 10.1016/S0375-9601(02)01411-1

[B45] Tickle-Degnen L. Rosenthal R. (1990). The nature of rapport and its nonverbal correlates. *Psychol. Inq.* 1 285–293. 10.1207/s15327965pli0104_1

[B46] Webber C. L. Zbilut J. P. (1994). Dynamical assessment of physiological systems and states using recurrence plot strategies. *J. Appl. Physiol.* 76 965–973. 8175612 10.1152/jappl.1994.76.2.965

[B47] Wiltermuth S. S. Heath C. (2009). Synchrony and cooperation. *Psychol. Sci.* 20 1–5. 10.1111/j.1467-9280.2008.02253.x 19152536

[B48] Yasuma F. Hayano J. (2004). Respiratory sinus arrhythmia: why does the heartbeat synchronize with respiratory rhythm? *Chest J.* 125 683–690. 10.1378/chest.125.2.683 14769752

